# Polarized light use in the nocturnal bull ant, *Myrmecia midas*

**DOI:** 10.1098/rsos.170598

**Published:** 2017-08-30

**Authors:** Cody A. Freas, Ajay Narendra, Corentin Lemesle, Ken Cheng

**Affiliations:** Department of Biological Sciences, Macquarie University, Sydney, New South Wales 2109, Australia

**Keywords:** ants, polarized light, celestial compass, route maintenance and nocturnal navigation

## Abstract

Solitary foraging ants have a navigational toolkit, which includes the use of both terrestrial and celestial visual cues, allowing individuals to successfully pilot between food sources and their nest. One such celestial cue is the polarization pattern in the overhead sky. Here, we explore the use of polarized light during outbound and inbound journeys and with different home vectors in the nocturnal bull ant, *Myrmecia midas*. We tested foragers on both portions of the foraging trip by rotating the overhead polarization pattern by ±45°. Both outbound and inbound foragers responded to the polarized light change, but the extent to which they responded to the rotation varied. Outbound ants, both close to and further from the nest, compensated for the change in the overhead e-vector by about half of the manipulation, suggesting that outbound ants choose a compromise heading between the celestial and terrestrial compass cues. However, ants returning home compensated for the change in the e-vector by about half of the manipulation when the remaining home vector was short (1−2 m) and by more than half of the manipulation when the remaining vector was long (more than 4 m). We report these findings and discuss why weighting on polarization cues change in different contexts.

## 1. Background

Arthropods are known to derive compass information using the pattern of polarized skylight [1–9]. Polarized light comprises light waves in which the wave occurs along a single plane. Light scatters after entering the earth's atmosphere and becomes partially linearly polarized. This creates an e-vector pattern in the sky arranged in concentric circles around the sun or moon's position [10,11]. The e-vector in the overhead sky remains in a stable orientation pattern perpendicular to the direction of the sun/moon. This stability makes the sky's polarization pattern a useful directional cue especially when the sun or moon's position is obscured [8,9,12–16]. Insects detect this polarized light through specialized photoreceptors that are located in the dorsal rim area of the eye [2,15–19].

Solar polarization is present even after sunset until the end of astronomical twilight when the sun's position passes 18° below the horizon [20]. During the evening or morning twilight, when the sun is near the horizon, the polarization pattern of the sky intensifies and simplifies along the North–South axis [21], making it of great interest to understand how animals that are active during twilight use this compass cue [3,5,9,12,16]. Among ants, there has been only one study [6] conducted on twilight-foraging animals [22,23]. In this study, outbound foragers of *Myrmecia pyriformis* confronted with a change in the polarization pattern by ±45° to the ambient pattern, modified their orientation, but only partially (17.96°). The authors suggested that this partial reliance was due to the extreme reliance on familiar visual landmarks that these ants exhibit [6,24]. Here, we investigate this further in a related nocturnal ant, *Myrmecia midas*, whose navigational capabilities have only recently been studied [25], in order to identify whether foragers use the pattern of polarized skylight during both the outbound and inbound journeys. We further explore whether the extent to which ants rely on polarized light changes with distance from the nest or length of the home vector during both outbound and inbound journeys.

## 2. Methods

**Figure 1.**
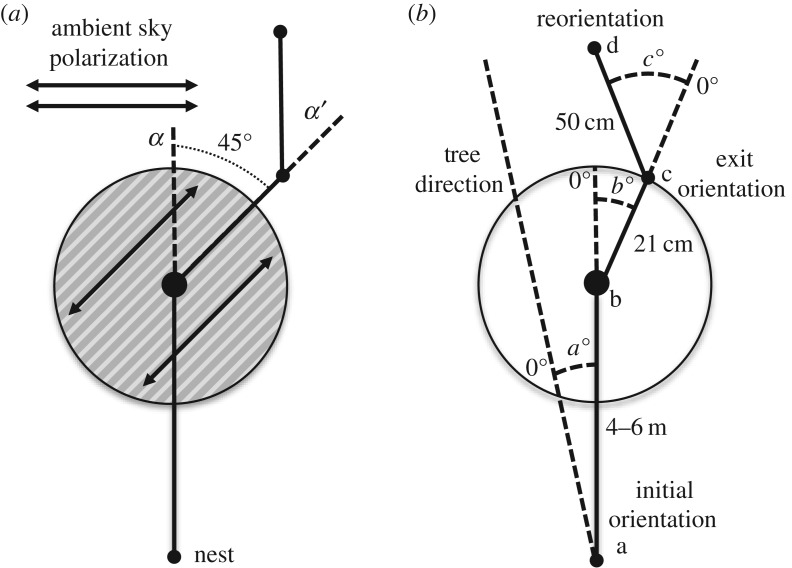
Schematics of the polarization filter and experimental set-up. (*a*) Diagram of the polarization filter. During the forager's outbound trip to the foraging tree, a polarization filter was placed over the forager with the polarization e-vector rotated ±45° of the ambient e-vector. This filter apparatus was used in a previous study [6]. (*b*) Diagram of measurements collected during polarization filter test. Measurements were made using a compass application on a smartphone. Initial orientation routes were measured from the nest entrance (a) to when the polarization filter was centred over the forager (b). Initial route directions (*a*°) were calculated with the tree direction from the nest as 0°. The magnitude of angle *a* has been artificially enlarged in this diagram for clarity, with angle *a* averaging 4.42° across all conditions during testing. Exit orientations were measured from the centre of the polarization filter (b) to the exit location of the ant on the filter's edge (c). Route directions under the filter (*b*°) were calculated from the forager's initial route direction. Reorientations were measured from the forager's exit location from the polarization filter (c) to the forager's path 50 cm after exiting the filter (d). Reorientation route directions (*c*°) were calculated from the under-filter route direction.

Experiments were conducted from September 2015 to November 2016 on two *M. midas* nests located on the northern portion of the Macquarie University North Ryde campus in Sydney, Australia (33°46′11′′ S, 151°06′40′′ E). *Myrmecia midas* nests were found in habitats consisting of stands of *Eucalyptus* trees with mostly barren understoreys with the nest entrance located near the base of a tree. Nocturnal foraging activity in this species [25] required the use of red-filtered headlamps in order to observe the ants. Research in ants does not require animal ethical approval within Australia. We modified the pattern of polarized skylight by rotating a polarization filter (42 cm diameter) above the ants. The polarization filter (Polaroid HN22; figure 1) was held by a circular 2 cm thick metal ring and lifted 10 cm off the ground by four equally spaced thin metal legs. Numbers of ants tested in each condition are given in the data figures. All testing was conducted during either the evening or morning twilight when the sun's position was between –18° and 0° relative to the horizon. Evening testing began 10 min after sunset and ceased before twilight ended. Morning testing began after the beginning of twilight and ceased before dawn. Each night we obtained the sun's position at sunset and sunrise from the Astronomical Almanac (http://asa.usno.navy.mil) and set the ambient e-vector 180° from this direction. As *M. midas* maintain predictable nest-foraging tree route patterns in a well-defined corridor, we were able to pinpoint the orientation of the overhead e-vector and rotate the polarizer relative to that direction. We relied on a compass to locate the ambient e-vector and rotate the polarizer by ±45° from this direction. When placing the polarized filter over the forager, we rested the compass on the polarizer along the filter's polarization pattern during placement. Only after the placement was confirmed did we remove the compass.

### 2.1. Outbound ants at different distances from the nest

We tested foragers at two distances, 4–6 m and 1–2 m from the nest. For the 4–6 m group, we chose foragers from two nests (Nest 1 and Nest 2), where some foragers travelled 12.8 m and 14.0 m from the nest to their foraging trees. For the 1–2 m group, we chose a separate group of foragers from Nest 1 that travelled 3 m to a foraging tree. We followed each forager and placed the centre of the polarizer over the ant when it was at 4–6 m or 1–2 m from the nest. In both conditions, the e-vector axis of the filter was oriented either ±45° relative to the dominant ambient polarization pattern (figure 1*a*), a method adapted from Reid *et al*. [6]. For each individual, we recorded the initial orientation, the exit orientation and their reorientation by placing small pegs in the ground (as defined in figure 1*b*). After a forager's positions were recorded, the forager was collected and marked with a small amount of enamel paint (Tamiya™, Japan) to ensure animals were not tested again. These marked foragers were then returned to the nest site.

### 2.2. Inbound ants at different distances from the nest

We tested inbound foragers at the same two distances (4–6 m and 1–2 m) from the nest. We followed foragers from Nest 1 travelling either 14 m (4–6 m condition) or 3 m (1–2 m condition) to their foraging tree during evening twilight. As a forager climbed the foraging tree, they were each collected in a plastic phial. Each forager was offered a small amount of honey and was then stored overnight in the dark (9 h). Each collected ant was marked with a small amount of enamel paint to exclude previously tested individuals. Foragers were released at the base of their foraging tree in the pre-dawn twilight, which corresponds to the time at which they typically return home [25]. We followed each ant as it travelled to the nest, and placed the centre of the polarizer on the ant when it reached a distance of 4–6 m or 1–2 m from the nest. Therefore, both inbound and outbound foragers were tested at the same distance from the nest. Similar to the outbound tests, the e-vector axis of the filter was oriented either ±45° relative to the dominant ambient polarization pattern. We recorded the initial orientation, the exit orientation and the reorientation of each forager in the same manner as in the outbound tests (figure 1*b*). Foragers were then followed for the remainder of their inbound path to ensure they returned to the nest site.

### 2.3. Conflict between home-vector length and nest location

Here, we tested individual foragers close to their nest but with a large remaining vector. We achieved this by first following foragers from Nest 1 to the foraging tree (14 m) in the evening twilight and collected them in a phial as they reached the foraging tree. Just as in previous inbound conditions, these foragers were fed, marked with paint, held overnight and released in the pre-dawn twilight. We released the foragers on the route at the halfway point between the nest and the foraging tree (7 m). Released foragers were allowed to return to 1–2 m from the nest entrance where the centre of the polarizer was placed over the ant. As with all previous conditions, the e-vector axis of the filter was either ±45° relative to the ambient e-vector. Identical to previous conditions, we recorded initial orientation, exit orientation and reorientation for each forager (figure 1*b*). Foragers were then followed for the remainder of their inbound path to record their final destination.

### 2.4. Statistical analysis

Data were analysed with circular statistics [26,27] using the statistics package Oriana Version 4 (Kovach Computing Services, UK). As each ant had a different initial heading direction, we corrected this by designating the initial heading as 0° for each animal. The *shift magnitude* of each path was calculated by taking the mirror of the difference between the forager's initial path direction and the forager's exit orientation in each −45° condition. This calculation allowed us to compare path shifts in both directions in degrees. Foragers' shift magnitudes were compared between the ±45° and between the two distance groups using Watson–Williams *F*-tests. If shift magnitudes between the two groups do not differ, then it means both groups rely on polarized light to the same degree. A Pearson's correlation coefficient was used to test the association between the lunar phase (in per cent) and shift magnitude under the filter. Lunar phase data were obtained from calculations in the Astronomical Almanac (http://asa.usno.navy.mil).

## 3. Results

### 3.1. Outbound ants at different distances from the nest

When the polarization filter was placed on an outbound ant at both testing distances, they initially stopped moving and then slowly began to move in a chosen direction. Most foragers would again stop as they reached the edge of the filter and performed visual scans before continuing on their chosen path. Ants did not pause after exiting the filter and continued on route towards their foraging tree.

#### 3.1.1. Outbound foragers at the 4–6 m distance

**Figure 2.**
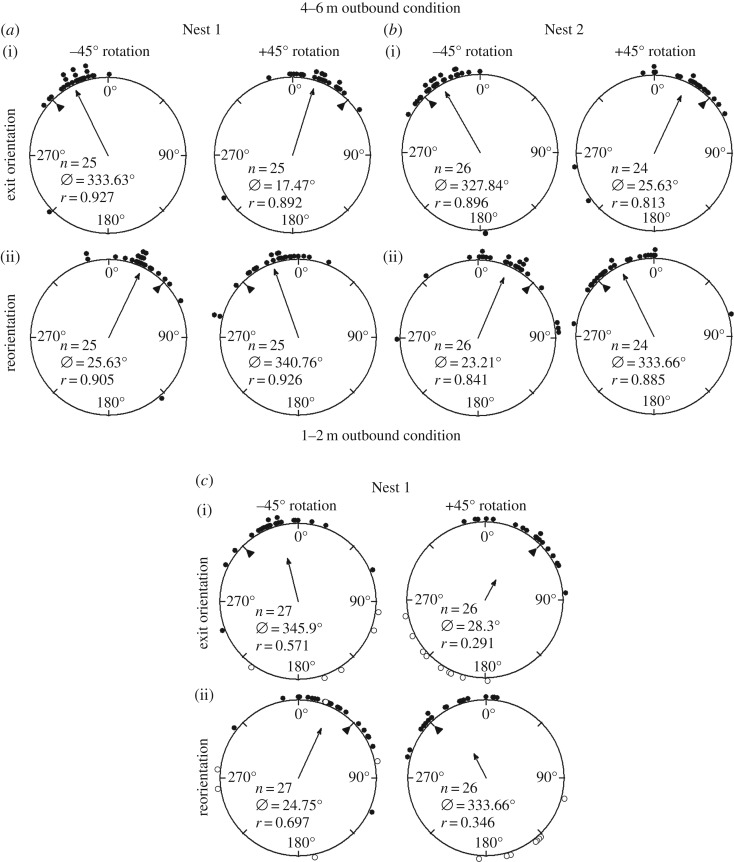
Circular distributions of individual *M. midas* foragers' headings during outbound conditions. Histograms show raw data of exit orientation under the filter and the reorientation after exiting the filter. The triangle denotes 45° in each distribution. The arrow denotes the length of the mean vector and the mean direction. (*a*) Orientations for Nest 1 during the 4–6 m outbound condition. (*b*) Orientations for Nest 2 during the 4–6 m outbound condition. (*c*) Orientations for Nest 1 during the 1–2 m outbound condition. Closed circles indicate individuals that continued on to the forging tree after testing. Open circles represent foragers that retreated once the filter was placed overhead and these individuals returned to within 30 cm of the nest entrance after testing. *n*, number of individuals; *Ø*, mean vector; *r*, length of the mean vector.

When the polarizer was rotated left (–45°), the ants' exit orientations were to the left of their initial direction of orientation (mean ± s.e.m.; Nest 1: *θ* = –26.37 ± 4.72°; Nest 2: *θ* = –32.16 ± 5.26°; table 1 and figure 2*a*(i)*,b*(i)), and these changes were significant at both nests (Watson–Williams *F*-test, Nest 1: *F* = 22.01, p≪0.01; Nest 2: *F* *=* 13.74, p≪0.01). Conversely, when the polarizer was rotated right (+45°), the foragers’ exit orientations were to the right of their initial heading direction (mean ± s.e.m.; Nest 1: *θ* = 17.47 ± 5.47°; Nest 2: *θ* = 25.07 ± 7.46°; table 1 and figure 2*a*(i)*,b*(i)). These changes were also significant at both nests (Watson–Williams *F*-test, Nest 1: *F* = 13.74, p≪0.01; Nest 2: *F* *=* 9.62, p≪0.01). After exiting the –45° rotated filter, foragers reoriented significantly to the right (Watson–Williams *F*-test, Nest 1: *F* *=* 18.25, p≪0.01, mean ± s.e.m. *θ* = 25.63° ± 5.11°; Nest 2: *F* *=* 9.65, p≪0.01, mean ± s.e.m. *θ* = 23.205 ± 6.57°; table 1 and figure 2*a*(ii)*,b*(ii)). After exiting the +45° rotated filter the foragers reoriented significantly to the left (Watson–Williams *F*-test, Nest 1: *F* *=* 12.57, p≪0.01, mean ± s.e.m. *θ* = –19.24 ± 5.76°; Nest 2: *F* *=* 5.83, *p* = 0.02, mean ± s.e.m. θ = –26.34 ± 5.26°; table 1 and figure 2*a*(ii)*,b*(ii)). Results did not differ between nests (*p* > 0.05 for both filter exit orientations and foragers' reorientations), and shift magnitude under the filter was not significantly different between the –45° and +45° conditions (Watson–Williams *F*-test, Nest 1: *F* *=* 0.17, *p* = 0.68; Nest 2: *F* *=* 0.194, *p* = 0.66).

**Table 1.** Statistics of outbound and inbound forager shifts under polar filter and reorientations.

	mean vector	95% CI		mean vector length	Rayleigh test	
condition	*μ* (°)	minus (°)	plus (°)	*r*	*Z*	*p*
*outbound 4–6* *m*						
Nest 1						
exit orientation						
−45°	333.63	324.86	342.40	0.927	21.46	<0.0001
+45°	17.47	6.5	28.2	0.892	19.89	<0.0001
reorientation						
−45°	25.63	15.61	35.66	0.905	20.47	<0.0001
+45°	340.76	331.94	349.50	0.926	21.45	<0.0001
Nest 2						
exit orientation						
−45°	330.3	320.05	340.55	0.896	20.92	<0.0001
+45°	25.03	10.44	39.62	0.813	15.88	<0.0001
reorientation						
−45°	23.21	10.33	36.08	0.841	18.41	<0.0001
+45°	333.66	322.36	344.96	0.885	18.81	<0.0001
*outbound 1–2* *m*						
exit orientation						
−45°	345.90	321.71	10.10	0.571	8.81	<0.0001
+45°	28.25	336.79	79.71	0.291	2.28	0.102
reorientation						
−45°	24.75	6.37	43.13	0.697	13.01	<0.0001
+45°	333.66	290.04	17.27	0.346	3.11	0.043
*outbound 1–2* *m non-retreaters*						
exit orientation						
−45°	346.47	334.18	358.76	0.897	14.50	<0.0001
+45°	32.81	20.26	45.37	0.893	14.36	<0.0001
reorientation						
−45°	23.36	10.17	36.55	0.883	14.03	<0.0001
+45°	330.56	319.07	342.06	0.91	14.90	<0.0001
*inbound 4–6* *m*						
exit orientation						
−45°	318.84	307.03	330.64	0.940	12.38	<0.0001
+45°	34.13	26.27	42.00	0.973	13.256	<0.0001
reorientation						
−45°	35.29	20.24	50.33	0.904	11.54	<0.0001
+45°	319.82	306.80	332.84	0.928	12.05	<0.0001
*inbound 1–2* *m*						
exit orientation						
−45°	335.14	326.62	343.66	0.955	14.592	<0.0001
+45°	19.73	10.16	29.30	0.957	13.73	<0.0001
reorientation						
−45°	27.59	15.31	39.86	0.909	14.592	<0.0001
+45°	332.65	320.65	344.64	0.933	13.06	<0.0001
*inbound vector and landmark conflict*						
exit orientation						
−45°	324.23	313.75	334.71	0.953	12.28	<0.0001
+45°	39.42	24.20	54.64	0.902	11.40	<0.0001
reorientation						
−45°	36.49	24.31	48.67	0.936	12.28	<0.0001
+45°	327.12	311.87	342.37	0.902	11.39	<0.0001

#### 3.1.2. Outbound foragers at the 1–2 m distance

**Figure 3.**
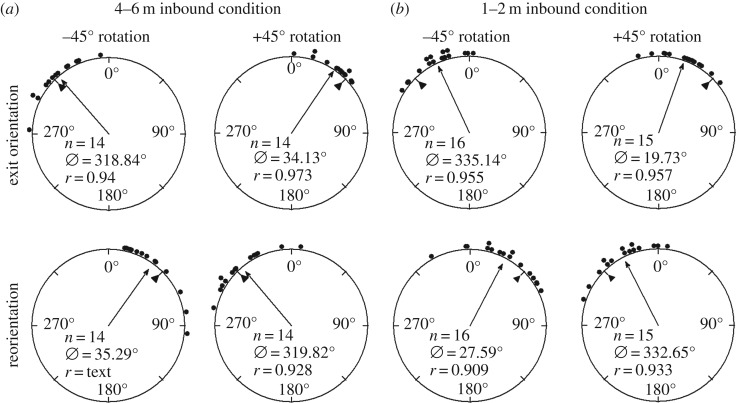
Circular distributions of individual *M. midas* foragers' headings during the inbound conditions. Histograms show raw data of exit orientation under the filter with the individual's initial orientation and reorientation with the forager's exit orientation under the filter. The triangle denotes 45° in each distribution. The arrow denotes the length of the mean vector and mean direction. (*a*) Orientations for Nest 1 during the 4–6 m inbound condition. (*b*) Orientations for the 1–2 m inbound condition. *n*, number of individuals; *Ø*, mean vector; *r*, length of the mean vector.

When the polarizer was rotated ±45°, individuals paused after the polarizer was placed overhead. After this short pause, most individuals continued to the foraging tree (+45°, *n* = 18; –45°, *n* = 22; figure 2*c* closed circles); a minority of individuals in both conditions, however, turned back and retreated (defined as individuals that returned to within 30 cm of the nest entrance after exiting the filter) to the nest after the polarizer was placed over them (+45°, *n* = 8; −45°, *n* = 5; figure 2*c* open circles). Focusing on only those individuals that continued to the foraging tree, when the polarizer was rotated 45° to the left (–45°), the foragers’ exit-orientations leaving the filter were to the left of their initial path direction (mean ± s.e.m.; Nest 1: *θ* = −18.26 ± 6.56°; table 1 and figure 3). This path change under the filter was significant (Watson–Williams *F*-test, Nest 1: *F* *=* 4.31, *p* = 0.04). When the polarizer was rotated 45° to the right (+45°), forager exit orientations were to the right of their initial path (mean ± s.e.m.; Nest 1: *θ* = 32.81 ± 6.4°; table 1 and figure 3) and this shift was also significant (Watson–Williams *F*-test, Nest 1: *F* *=* 12.29, p≪0.01). After exiting the –45° rotated filter the foragers reoriented significantly to the right (Watson–Williams *F*-test, Nest 1: *F* *=* 9.95, p≪0.01, mean ± s.e.m. *θ* = 26.23 ± 6.73°; table 1 and figure 2*c*), and after exiting the +45° rotated filter the foragers reoriented significantly to the left (Watson–Williams *F*-test, Nest 1: *F* *=* 10.79, p≪0.01, mean ± s.e.m. *θ* = –29.43 ± 5.87°; table 1 and figure 2*c*). While the number of individuals who retreated was insufficient for statistical analysis, foragers' exit orientations shifted as would be expected, either to the left (mean ± s.e.m. *θ* = –35.34 ± 26.60°) or to the right (mean ± s.e.m. *θ* = 37.14 ± 9.33°) of the nest entrance direction corresponding with manipulations in the polar filter (–45° or +45°, respectively). Shift magnitude was not significantly different between the –45° and +45° conditions (Watson–Williams *F*-test; 0.65, *p* = 0.43). When the –45° and +45° shifts were combined, the shift magnitude was also not significantly different between the outward heading ants of the two outbound testing conditions (Watson–Williams *F*-test, *F* *=* 0.17, *p* = 0.68).

### 3.2. Inbound ants at different distances from the nest

When foragers were released back at the base of their foraging tree during the morning twilight, they also initially paused for a brief period and scanned the environment without translation, before travelling in the nest direction. Nest-bound foragers typically paused again once the polarization filter was placed above them, yet some individuals continued their forward movement. The same behavioural difference occurred at the filter edge, as some inbound foragers did not stop at the edge of the polarizer. After exiting the filter, all foragers continued on to the nest entrance and entered the nest.

#### 3.2.1. Inbound foragers at the 4–6 m distance

When the polarizer was rotated to the left (–45°), the foragers’ exit orientations were to the left of their initial direction of orientation (mean ± s.e.m.; Nest 1: *θ* = –41.16 ± 6.02°; table 1 and figure 3*a*), and these changes were significant (Watson–Williams *F*-test, Nest 1: *F* *=* 39.17, p≪0.01). When the polarizer was rotated to the right (+45°), foragers' exit orientations were to the right of their initial heading direction (mean ± s.e.m.; Nest 1: *θ* = 34.13 ± 4.01°; table 1 and figure 3*a*), and these changes were significant (Watson--Williams *F*-test, Nest 1: *F* *=* 50.57, p≪0.01). After exiting the –45° rotated filter, the foragers reoriented significantly to the right (Watson--Williams *F*-test, Nest 1: *F* *=* 29.07, p≪0.01 mean ± s.e.m. *θ* = 35.29 ± 6.02°; table 1; figure 3*a*). After exiting the +45° rotated filter the foragers reoriented significantly to the left (Watson--Williams *F*-test, Nest 1: *F* *=* 62.51, p≪0.01, mean ± s.e.m. *θ* = –40.18 ± 6.64°; table 1; figure 3*a*). Shift magnitude under the filter was not significantly different between the –45° and +45° conditions (Watson–Williams *F*-test, *F* = 1.12, *p* = 0.301).

#### 3.2.2. Inbound foragers at the 1–2 m distance

When the polarizer was rotated to the left (–45°), the foragers’ exit orientations were to the left of their initial direction of orientation (mean ± s.e.m.; Nest 1: *θ* = –24.86 ± 4.35°; table 1 and figure 3*b*), and these changes were significant (Watson–Williams *F*-test, Nest 1: *F* *=* 23.51, p≪0.01). When the polarizer was rotated to the right (+45°), foragers' exit orientations were to the right of their initial heading direction (mean ± s.e.m.; *θ* = 19.73 ± 4.88°; table 1 and figure 3*b*), and these changes were significant (Watson–Williams *F*-test, *F* *=* 18.59, p≪0.01). After exiting the –45° rotated filter the foragers reoriented significantly to the right (Watson–Williams *F*-test, *F* *=* 20.84, p≪0.01, mean ± s.e.m. *θ* = 27.59 ± 4.35°; table 1 and figure 3*b*). After exiting the +45° rotated filter, the foragers reoriented significantly to the left (Watson–Williams *F*-test, Nest 1: *F* *=* 21.25, p≪0.01, mean ± s.e.m. *θ* = –27.35 ± 6.12°; table 1 and figure 3*b*). Shift magnitude under the filter was not significantly different between the –45° and +45° conditions (Watson–Williams *F*-test, *F* = 065, *p* = 0.43). When the –45° and +45° shifts were combined, total shift magnitude in foragers tested at 1–2 m was significantly smaller than foragers tested at 4–6 m (Watson–Williams *F*-test, *F* *=* 10.93, p≪0.01).

### 3.3. Conflict between home-vector length and nest location

**Figure 4.**
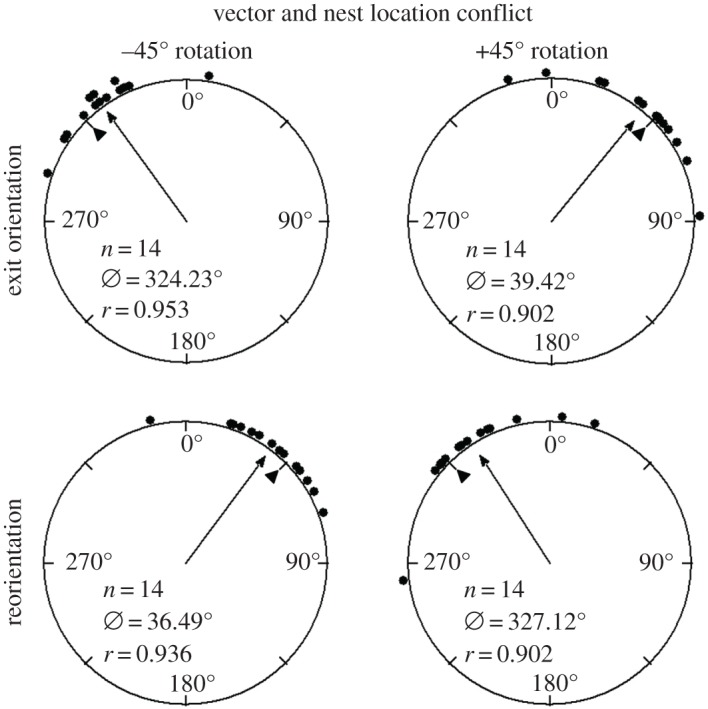
Circular distributions of individual *M. midas* foragers' headings during the long-vector 1–2 m inbound condition. Histograms show raw data of exit orientation under the filter with the individual's initial orientation and reorientation with the forager's exit orientation under the filter. The triangle denotes 45° in each distribution. The arrow denotes the length of the mean vector direction. *n*, number of individuals; *Ø*, mean vector; *r*, length of the mean vector.

Inbound ants with 14 m home vectors were displaced on the route but half way home and had to travel only 7 m to find the nest. The ability of these ants to detect a change in the pattern of the polarized light was assessed at 1–2 m from the nest entrance. When the polarizer was rotated to the left (–45°), the foragers’ exit orientations were to the left of their initial direction of orientation (mean ± s.e.m.; Nest 1: *θ* = –35.77 ± 5.35°; table 1 and figure 4), and these changes were significant (Watson–Williams *F*-test, Nest 1: *F* *=* 50.78, p≪0.01). When the polarizer was rotated to the right (+45°), foragers' exit orientations were to the right of their initial heading direction (mean ± s.e.m.; *θ* = 39.42 ± 7.77°; table 1 and figure 4), and these changes were significant (Watson–Williams *F*-test, *F* *=* 29.09, p≪0.01). After exiting the –45° rotated filter, the foragers reoriented significantly to the right (Watson–Williams *F*-test, *F* *=* 37.59, p≪0.01, mean ± s.e.m. *θ* = 36.49 ± 6.21°; table 1 and figure 4). After exiting the +45° rotated filter, the foragers reoriented significantly to the left (Watson–Williams *F*-test, Nest 1: *F* *=* 15.67, p≪0.01, mean ± s.e.m. *θ* = –32.88 ± 7.78°; table 1 and figure 4). Shift magnitude size was not significantly different between the –45° and +45° conditions (Watson–Williams *F*-test, *F* = 0.18, *p* = 0.677). Foragers in this condition showed shift magnitude size similar to that of foragers tested 4–6 m from the nest entrance (Watson–Williams *F*-test, F≪0.01, *p* = 0.99), and these combined shift magnitudes were significantly greater than foragers tested at the 1–2 m vector travelling from a tree 3 m away (Watson–Williams *F*-test, *F* *=* 8.35, p≪0.01).

### 3.4. Lunar phase

Across all conditions, lunar phase was not associated with changes in shift magnitude of foragers while under the filter (Pearson's correlation coefficient, *r* = −0.127, *p* = 0.074).

## 4. Discussion

In this study, both inbound and outbound foragers changed their heading direction in response to changes in the overhead polarization pattern. In outbound foragers, we found that distance away from the nest did not influence the weighting foragers gave to this cue. Conversely, ants rely most on the pattern of the polarized skylight when they are returning home (inbound) and have a long accumulated vector (4–6 m).

In *M. pyriformis*, use of the polarization cue was tested only in outbound ants close to the nest [6]. Here, when the polarized filter was rotated by ±45°, ants changed their heading direction in the appropriate direction but by less than half of the rotation (–21.8° for –45° rotation; +14.1° for +45° rotation). In our study, the outbound *M. midas* ants at both 1–2 m and 4–6 m away from the nest (figure 2) compensated for the change in the overhead e-vector by about half of the manipulation (1–2 m group: –18.26° for –45°; 32.81° for +45°; 4–6 m group: −26.37° (Nest 1) and −32.16° (Nest 2) for –45°; 17.47° (Nest 1) and 25.07° (Nest 2) for +45°). Both species appear to choose a compromise heading direction between the celestial and terrestrial compass cues during their outbound journey, and this appears to hold true at different distances from the nest.

In the inbound condition, we found that foragers of *M. midas* tested 1–2 m from the nest compensated for the change in the overhead e-vector by about half of the manipulation in their heading (–24.86° for –45° and 19.73° for +45°, figure 3*a*). Interestingly, unlike in the outbound conditions, inbound *M. midas* ants tested at 4–6 m from the nest compensated for well over half of the e-vector manipulation in their altered heading (–41.16° for –45° and 34.13° for +45°, figure 3*b*). Such large compensation was also found in inbound foragers that had a 14 m home vector but were released half way home and tested close (1–2 m) to the nest (–35.77° for –45° and 39.42° for +45°, figure 4). This shows that inbound ants respond more to a change in the pattern of polarized skylight than outbound ants. These results imply that inbound ants weight polarization cues differently: ants with a longer home vector respond more to a change in the polarization pattern.

Our results suggest that foragers use both terrestrial and celestial cues, but the weighting of these cues appears to change with the ant's foraging context. In this study, *M. midas* foragers en route appear to weight vector cues in combination with the surrounding terrestrial cues, shifting their paths significantly under an altered polarization pattern. Yet when *M. midas* foragers are displaced to a local area with a vector direction conflicting with the surrounding terrestrial cues, individuals ignore the accumulated vector and orient using only the terrestrial cues [25]. Thus, it appears that nocturnal *Myrmecia* ants use the pattern of the polarized skylight only when the readouts of the celestial and terrestrial cues align. Furthermore, when there is a conflict between the two sources of compass cues, the celestial cues are suppressed or ignored [25]. This further implicates navigational context as a factor in celestial cue weighting. These behavioural differences may arise as foragers in the polarization experiment encounter no mismatch in cue sets before the polarized light filter, causing them to respond to the altered polarized light pattern while under the filter. Whereas after displacements off-route, foragers are presented with a mismatch between the familiar visual territory and their stored views, causing them to ignore celestial compass information when returning home [25].

The significant differences in shift magnitude in inbound foragers under different conditions were not predicted. Inbound foragers travelling from long distances (14 m, longest foraging route at this site) show larger shifts under the filter compared with individuals that forage in trees closer to the nest regardless of proximity to the nest. These disparities suggest greater weight is being placed on the polarization compass when in conflict with terrestrial cues in these foragers. It appears that the proximity of the nest tree at the test location, a potentially salient terrestrial cue, does not decrease the observed shifts in these far-foraging, long-vector individuals, implying that vector length clearly influences the weight given to the polarization pattern cue. These increases align with the hypothesis that with longer accumulated vectors, ants put more weight on these vector cues [28]. In our case, this difference in weighting persists even after a 9 h delay, with the direction of polarized light, linked to the position of the sun, having changed. These delay periods align with this species' foraging ecology as foragers typically spend this period on their foraging tree overnight [25]. Our results also align well with those from our previous *M. midas* study where only long-vector (more than 5 m) individuals show any evidence of orientation using path integration after distant displacement [25]. It may also be possible that, as tree fidelity has not been studied in this species, there may be some other difference between individuals that forage further from the nest site and those that forage at a nearer tree. These differences could include disparities in visual scenes encountered by these two foraging groups at Nest 1 or potentially even genetic differences between foragers travelling different distances to the nest. Further study into these behavioural choices is merited to untangle these possibilities.

Furthermore, it is interesting that the large shifts in long-vector inbound foragers are not seen in outbound foragers travelling to the same foraging tree. Vector memories in these outbound foragers are based on past foraging trips, whereas inbound foragers are using the vector memory of the current foraging trip [29,30]. This discrepancy may influence the weight these individuals give the vector cue. Unfortunately, as Nest 2 has since died, our field site currently has only one known nest with individuals foraging long distances (more than 5 m), making study of these differences difficult. Further study into this species and its use of celestial cues for navigation is warranted.

It is worth noting that the observed heading directions in both outbound and inbound foragers could be in part due to visual changes caused by the filter, independent of the e-vector rotation. Beyond the e-vector shift, light intensity levels are reduced, and there are changes in the visibility and salience of both celestial and terrestrial cues under the filter. These changes could alter the weighting of cues in this study compared to foragers navigating under natural conditions.

## 5. Conclusion

We show that both outbound and inbound *M. midas* foragers respond to changes of the e-vector orientation. Outbound ants compensate only partially to the change in polarized light, and this holds true at different distances from the nest. Inbound foragers with a longer home vector respond almost fully to the change in the pattern of the polarized skylight.

## Supplementary Material

FreasC_DataSet_ESM

## Data accessibility

Our data are collected in the electronic supplementary material.
